# Using Three Cross-Sectional Surveys to Compare Workplace Psychosocial Stressors and Associated Mental Health Status in Six Migrant Groups Working in Australia Compared with Australian-Born Workers

**DOI:** 10.3390/ijerph16050735

**Published:** 2019-02-28

**Authors:** Alison Daly, Renee N. Carey, Ellie Darcey, HuiJun Chih, Anthony D. LaMontagne, Allison Milner, Alison Reid

**Affiliations:** 1 School of Public Health, Curtin University, Bentley, WA 6102, Australia; alison.daly@curtin.edu.au (A.D.); renee.carey@curtin.edu.au (R.N.C.); h.chih@curtin.edu.au (H.C.); 2 Centre for Genetic Origins of Health and Disease, School of Biomedical Science, Curtin University and The University of Western Australia, Perth, WA 6000, Australia; ellie.darcey@uwa.edu.au; 3 Centre for Population Health Research, Deakin University, Burwood, VIC 3125, Australia; tony.lamontagne@deakin.edu.au; 4 Centre for Health Equity, School of Population and Global Health, University of Melbourne, Melbourne, VIC 3010, Australia; allison.milner@unimelb.edu.au

**Keywords:** migrant workers, workplace psychosocial stressors, mental health, cross-sectional surveys

## Abstract

Migrant workers may be more likely to be exposed to workplace psychosocial stressors (WPS) which have an affect on physical and mental health. Given the relative lack of research on this topic, the study objectives were to estimate and compare the prevalence of WPS in migrant and Australian workers and investigate associated mental health problems. Three cross-sectional surveys, two with migrant workers and one with Australian workers, were pooled to provide estimates of prevalence. Regressions were conducted to investigate associations between workers and WPS. All WPS, except unfair pay, were associated with higher probability of mental health problems. The association between WPS and mental health did differ between some migrant groups. Compared with Australian-born workers, all other migrant groups tended to have a lower risk of mental health outcomes. Interactions between WPS and migrants showed variable levels in the risk of having a mental health problem, some attenuated and some increased. The study showed that country of birth does play a part in how treatment in the workplace is perceived and responded to. Any interventions to improve workplace conditions for migrant workers need to be aware of the different experiences related to migrant ethnicity.

## 1. Introduction

The search for decent work, for many, is the most important driver of migration [1]. Workers leave their home countries to improve their quality of life and to improve their employment opportunities and circumstances in their new host countries. They may migrate temporarily or permanently as skilled or unskilled workers, with or without a legal right to enter and work in the host country. Furthermore, many migrants’ right of stay in the host country is contingent on the good opinion of an employer. Migrant workers, therefore, tend to cluster at the top and bottom of occupational hierarchies in their host countries and their working and occupational health and safety conditions can, therefore, vary widely. Research from several countries showed that migrant workers experience higher work-related injuries and fatalities than native-born workers [2,3] and are more likely to be exposed to workplace hazards, including carcinogens [4,5]. This is largely because the majority of migrant workers undertake poorer-quality jobs where exposure to hazards is more likely, although work from Australia showed that workplace exposure to carcinogens was greater among ethnic minority than native-born workers, even within the same jobs [4].

Even workers at the top of the occupational hierarchy are not immune to experiencing workplace psychosocial stressors [6,7]. By these, we mean factors such as working in jobs in which you have low control, working in jobs with high demand and complexity, working in jobs that are not paid fairly, burnout [8,9,10], or jobs that have low security or high job strain [11]. Evidence from both cross-sectional and longitudinal studies shows that these factors in any workers are associated with poor mental [8,12,13,14,15,16,17,18], and physical health [19,20,21,22,23,24], and increased absence from work [25,26].

Migrants are generally healthier than the population they migrate to, although over time their health was found to regress to the levels of the host population [27]. Experiencing adverse conditions at work and/or greater adverse conditions at work than the host population may be a feature that contributes to that health decline. Given the importance of work for many migrants and the relative lack of research on this topic, we aimed to investigate (1) the prevalence of exposure to workplace psychosocial stressors among migrants in comparison with Australian-born workers, and (2) the relationship between exposure to psychosocial job stressors and mental health outcomes among migrants (compared with Australian-born workers).

## 2. Materials and Methods

This study pools data that are common from three cross-sectional telephone surveys investigating workplace psychosocial stressors in Australian workers; two surveys targeted selected migrant groups, and one investigated Australian-born workers. For each, ethics approval was obtained from the Human Research Ethics Committee of Curtin University (HREC RDHS-55-16).

### 2.1. Sample

There is no accessible source of migrant workers’ contact information available in Australia. We attempted various sampling methods in these surveys to try and ensure some degree of random sampling while yielding the numbers necessary to provide statistical power to investigate any differences in exposure to workplace hazards between migrant and Australian-born workers.

The first survey, the Migrant Worker I study, conducted in 2013, recruited workers of Vietnamese, Chinese, and Arabic-speaking ancestry. To obtain the sample for this survey, the 2011 Census [28] was used to identify suburbs in the cities of Melbourne, Sydney, or Perth with a high concentration of residents of Arabic-speaking, Chinese, or Vietnamese background. This list of suburbs and a second list of the most common surnames for these groups was then provided to a commercial survey sampling firm who compiled a list of telephone numbers, including both land lines and mobiles, in those suburbs. Anyone aged 18 years to 65 years, currently employed, from one of a Vietnamese, Chinese, or Arabic-speaking background and living in Melbourne, Sydney, or Perth was eligible to participate. A total of 9898 households were contacted over the course of the study, of which 1448 had someone in the household who met the eligibility criteria. Of these, 585, a quota sample of 195 per ethnic group, consented to participate, giving a response rate of 40.4%.

The second survey, the Australian Worker study, conducted in 2017, recruited a random sample of Australian-born people of Caucasian ancestry, currently working and aged 18 to 65 years stratified by state and whether or not they lived in rural or metropolitan areas, using the latest version of the Electronic White Pages (EWP), which included both land line and mobile telephone numbers where listed. A total of 33,103 households were contacted, of which 1217 had someone in the household who met the eligibility criteria; of these, 1062 consented to participate, giving a response rate of 87.3%.

The third survey, the Migrant Worker II study conducted in 2017/2018, recruited people born in New Zealand, India, or the Philippines, who were aged 18 to 65 years and currently working. A variety of methods were necessary to obtain the desired sample size for each migrant worker group: the first used random sampling of the latest EWP, stratified by state and then filtered by the most common surnames for peoples born in the target countries; the second method refined the sample frame by only selecting suburbs that had high proportions of the target migrants; the third method procured samples from a commercial survey sampling firm which was able to identify members of the target migrant groups. In addition to these methods, a combination of snowballing and advertising was employed to try to find and recruit the desired number of Filipino workers. Using these methods, a total of 310,636 households were contacted, of which 2051 had someone in the household who met the eligibility criteria; of these, 1630 consented to participate giving a response rate of 79.5%. Table S1 (Supplementary Materials) shows the methods used and the numbers recruited by country of birth.

All interviews were conducted by trained interviewers using computer-assisted telephone interviews. Following a brief introductory script given in English, where consent to continue was obtained, participants whose first language was not English were given the option of completing the interview in Arabic, Vietnamese, Mandarin, Cantonese, Hindi, or Tagalog. For Arabic, Vietnamese, and Chinese language translations, a bilingual interviewer conducted the interview and direct translation of the questionnaire was done at the time of the interview. No one elected to have the interview conducted in Hindi or Tagalog.

Using the same basic questionnaire, all three surveys collected demographic information, including gender, country of birth, year of arrival in Australia, language most commonly spoken at home, and highest level of education. Year of birth was collected for the Migrant Worker I study; however, due to the number of missing values, this variable was changed to age range for the Migrant Worker II and Australian Worker studies. Information was also collected about employment, including type of contract (casual, permanent, or fixed term), number of hours worked, self-employed or not, company size, the industry of employment, and details about tasks undertaken at work. The Australian and New Zealand Standard Classification of Occupations (ANZSCO) was used to code occupation [29,30].

### 2.2. Measures

Psychosocial job quality was estimated from a questionnaire including questions about (a) high job demand (four questions: how stressful the job is; how complex and/or difficult the job is; whether or not new skills must be acquired; and whether or not existing skills are used); (b) low job control (three questions: whether or not the worker can determine how and when the work is done; and whether they have input into the job); (c) low job security (three questions: worry about security of job, and the future and security of the company); and (d) the balance of effort to reward (one question: perceived fairness of pay). These measures were previously validated [31]. These four factors were measured using a seven-point Likert scale, ranging from strongly agree to strongly disagree.

The summed responses to each psychosocial job quality factor were dichotomised using the 75th percentile as the cut point to assign respondents to high demand (indicating increasing demand), and the 25th percentile to assign respondents to low control, low security, and unfair pay categories (indicating decreasing control, security, and fair pay) [31]. Exposure to two or more of the workplace psychosocial stressors was defined as psychosocial job adversity [31].

The Migrant Worker I survey, conducted with workers from Vietnam, China, and Arabic-speaking countries, used the Mental Health Inventory (MHI5) to estimate the probability of having a mental health problem. The MHI5 was established as a valid instrument for assessing mental health in the general population [32]. Using a six-point Likert scale ranging from all of the time to none of the time, respondents rated the frequency of feeling a particular way, such as calm or depressed in the last four weeks, and responses were then summed for the total score. The total scores were standardised and ranged from 0 to 100, with higher scores indicating better mental health.

For the second and third surveys conducted with workers of Caucasian ancestry born in Australia, and workers born in New Zealand, India, and the Philippines, the Kessler 6 (K6) was used. The K6 measures psychological distress by asking how often people felt a particular way in the last four weeks on a five-point Likert scale ranging from all of the time to none of the time. The summed K6 scores ranged from 6–30 with higher scores indicating poorer mental health.

Both of these measures can be validly used as indicators of mental health including anxiety and depression, and a cross-walk study identified equivalent cut points [33]. Using this cross-walk, cut points less than or equal to 52 and greater than or equal to 15 for the MHI-5 and K6, respectively, were used to identify participants who were likely to have a mental health problem [34,35,36,37].

### 2.3. Analysis

The Migrant Worker I study had missing data for age (*n* = 71, 3.3%), for those born outside Australia, and for the year of arrival in Australia (*n* = 25, 1.2%), with a relatively even distribution across the three ethnic groups. These missing values were, therefore, assumed to be random. To enable comparable descriptive estimates, data from the three surveys were pooled for all common items and checked for consistent coding. The data were then weighted using iterative proportional fitting [38] by age, gender, and education for employed persons within each migrant group using the Australian Bureau of Statistics Census data that were closest to the year of the survey [28,39] The data were also weighted by area of residence within the state (metropolitan and rest of state) for the workers born in Australia, New Zealand, India, or the Philippines.

Univariate descriptive analysis produced estimates with 95% confidence intervals for socio-demographic and employment variables and exposure to psychosocial job adversity. To identify associations between migrant groups, psychosocial job quality factors, and overall job adversity, logistic regression was used. Unadjusted models included psychosocial job quality and estimates of mental health problems as dependent variables and migrant group as the independent variable. All the logistic regression models were then repeated using the variables in the univariate analysis as covariates. The interactions of ethnic group and psychosocial job quality factors in these models were used to assess the adjusted odds ratios for mental wellbeing by country of birth. Post-estimation tests were conducted for fit using contrast Hosmer–Lemeshow chi-square for logistic models [40] and scalar measures of fit for regression models [41]. A value of *p* < 0.05 was assumed to be statistically significant. All analyses were conducted using Stata V.14 [42].

## 3. Results

Almost half of the Migrant Worker I study respondents (49.4%) elected to be interviewed in a language other than English. Of these 289 respondents, 52% were of Vietnamese background, 35% were of Chinese background, and 13% were from Arabic-speaking countries. All other interviews were conducted in English. Ten percent of respondents from the Migrant Worker I study were born in Australia (13 people of Vietnamese ancestry, 11 people of Chinese ancestry, and 39 from Arabic-speaking countries). As they did not differ significantly from those born outside Australia in terms of age, gender, and job quality variables, they were included in this analysis within their ethnic groups.

There was a higher percentage of male workers born in India and New Zealand compared with workers born in Australia (Table 1). Workers born in India had the highest percentage of workers aged 26–35 years and the lowest percentage of workers aged 36–45 years compared with workers born in any other country except China, whereas a higher percentage of New Zealand workers were aged 56 years and older compared with workers from all other countries except India and Vietnam (Table 1).

Workers born in India and the Philippines were more likely to have completed tertiary education compared with workers born in any other country. In contrast, workers born in Vietnam, China, or an Arabic-speaking country were more likely to have had twelve years of schooling compared with workers born in other countries. Both Australia and New Zealand had higher percentages of workers with trade, diploma, and certificate qualifications compared with workers born in the other countries (Table 1).

A lower percentage of workers born in Australia had full-time employment compared with workers born in New Zealand and India. Workers born in New Zealand worked more hours per week on average than workers from any other country. A higher percentage of workers born in the Philippines were employed on a permanent basis compared with workers born in Australia, China, and Arabic-speaking countries. A higher percentage from these latter two countries reported working on a casual basis. Workers born in the Philippines were least likely to be self-employed compared with any other country (Table 1).

There were many differences in occupational categories across the different ethnic groups, but few were statistically significant. A notable exception was the high percentage of workers born in the Philippines who were working as labourers despite half reporting having completed tertiary education. Workers from India had the greatest percentage employed professionally.

Compared with Australian-born workers, Vietnamese workers were statistically significantly more likely to report being in high-demand jobs and less likely to report being in jobs with low control or low security (Table 2). Chinese workers were significantly more likely to report jobs with low control compared with workers from other countries. By contrast, Australian-born workers were significantly less likely to report having low-security jobs compared with workers from New Zealand, India, or the Philippines. Filipino and Vietnamese workers were significantly less likely to report jobs with unfair pay compared with Australian-born workers (Table 2).

After adjusting for demographic and employment variables, all psychosocial job quality factors, with the exception of unfair pay, were associated with the probability of having mental health problems across the whole sample (Table 3). Of note, workers from Arabic-speaking countries had a twofold increased risk of having a mental health problem, but there was no difference among the other migrant groups.

When the interaction between country of birth and the indicator of psychosocial job adversity was included in the model, country of birth was not uniformly associated with job quality factors in relation to mental health (Table 4).

Jobs with low control appear to affect workers born in Vietnam more than workers born in the other countries. Workers from Arabic-speaking countries had the highest risk of mental health problems when they felt they were unfairly paid compared with workers from any other country. Workers from Vietnam had lower risks of mental health problems when working in low-security jobs compared with Australian-born workers. Compared with Australian-born workers, having jobs with two or more measures of adversity did not significantly increase the risk of mental health problems for migrant workers overall (Table 4).

## 4. Discussion

Work is an important determinant of health and, for positive mental health, the type or quality of work is of more importance than having a job per se. Poor-quality jobs can be detrimental to workers’ mental health, and more detrimental than unemployment [11]. In this current study, we found disparities in the prevalence of working in jobs with adverse psychosocial factors between Australian-born and migrant workers. There was no strictly observable pattern; migrants reported a higher or lower prevalence across all factors compared with Australian-born workers. Overall job adversity, working in complex or demanding jobs, and jobs with low security were associated with probable mental health problems in all workers; however, in general, there were few statistically significant differences between groups, excepting workers from Arabic-speaking countries who had a twofold increased risk of mental health problems.

In this current study, we found the prevalence of working in a job with perceived low security differed across migrant groups. Workers from New Zealand, India, and the Philippines were not significantly more likely to report perceived low security, compared with Australian-born workers. In contrast, workers from Vietnam, China, and Arabic-speaking countries were less likely to report perceived low security than Australian-born workers. Job insecurity is a global occupational health problem with 49% of United States (US) workers worrying about the future stability of their job, as well as 46% of Spanish workers and 28% of workers in the Netherlands [43]. Other work from Spain found that immigrant workers had a higher prevalence of working in low-security jobs compared with native-born workers, (36% vs. 33%, respectively) [44].

In terms of the other measures of psychosocial job quality compared with Australian-born workers, workers from Vietnam reported an increased prevalence of working in complex or demanding jobs in this current study. Furthermore, workers from Vietnam had a lower prevalence, and workers from China a higher prevalence of working in jobs with low control. There were no disparities in the prevalence of unfair pay by migrant group. Similar findings were reported from a comparative study in Spain comparing psychosocial factors at work among migrant and native-born workers. Foreign-born workers (40.6%) had an increased prevalence of exposure to work with high quantitative demands compared with native-born workers (30.3%). In the same study there was an increase in the prevalence of low influence (47.0% vs. 37.7%) and low control of working hours (47.0% vs. 35.8%) among foreign-born workers compared with native-born workers. However, the Spanish study did not examine migrant groups separately by country of birth; thus, it is unclear whether some groups were more impacted than others [44]. More than one-third of workers from all ethnic groups reported unfair pay in our study. This is increasingly recognised as a problem for migrant workers in Australia, particularly temporary migrant workers, where a recent study found that one-third of all workers interviewed (*n* = 4322) were paid less than half the minimum wage ($12 or less) and 46% were paid $15 or less per hour. This did not appear to vary by nationality, as more than one-fifth of workers from all nationalities earned <$12 per hour [45].

Similar to findings from both cross-sectional and longitudinal studies [11,46,47], our study found that, across all workers, working in a low-security job was associated with a twofold increased risk of poorer mental health. Workers in the Netherlands reporting low job security had a twofold increased risk of mental health symptoms measured using the MHI-5 and after adjusting for sociodemographic variables and any 12-month physical disorder [46]. A similar pattern of negative mental health was observed among workers in 27 European countries, who reported working in insecure jobs [47]. Likewise, work examining seven waves of the Household, Income, and Labour Dynamics in Australia Survey (HILDA) found a twofold increased risk of mental health problems associated with working in a low-security job, after adjusting for a range of sociodemographic measures [11].

However, our study found that the impact on mental health from working in low-security jobs varied by migrant group. Compared with Australian-born workers, all other migrant groups tended to have a lower risk of mental health outcomes, but the differences were not statistically significant, although, for workers from Arabic-speaking countries, the difference approached significance (*p* < 0.052). It is unclear why this might be the case. Cultural and “traditional” values were shown to moderate the relationship between job insecurity and poor mental health. A study of workers in two factories undergoing job losses in the United States found that workers with collectivist values were more negatively impacted by job insecurity than workers with an individualistic orientation [48]. Similarly, workers who held more “traditional” values (e.g., fatalism, respect for authority) were more negatively impacted by job insecurity [49]. However, in our study, we found that workers from collectivist cultures (e.g., China, India, and the Philippines) appeared to be less impacted by job insecurity than Australian-born workers. This may be related to expectations of work security in their home country. Trade liberalization in all three countries, as a result of globalisation, led to instability in financial markets and subsequent changes in worker rights and tenure [50]. We did not collect information on working conditions in the home country. Another possible explanation may lie with the expectations of Australian-born workers. The move away from permanent/full-time work with benefits to more temporary and insecure work arrangements is occurring in Australia since the 1970s [51], and, in the past two decades, there was a rapid increase in the number of temporary workers, specifically workers on fixed-term contracts and those employed by temporary or labour-hire agencies [52]. This rapid transition may have resulted in an overwhelming sense of job insecurity among Australian workers. In our study, Australian-born workers had a similar prevalence of low-security jobs, but poorer mental health outcomes associated with low security compared with other migrant workers. This concurs with other work from Australia that showed that permanent full-time workers did not report the lowest perceived job insecurity of all workers [52].

The impact on mental health of the other psychosocial factors examined in this current study varied by migrant group. Across all workers, working in complex or demanding jobs was associated with close to a threefold increased risk of mental health problems, but this did not vary by migrant group. Across all workers, there was an increased risk of poorer mental health associated with working in jobs with low control, and Vietnamese workers had an eightfold increased risk of mental health problems associated with low control. Across all workers, unfair pay was not associated with poorer mental health, although for workers of Arabic-speaking background, there was double the risk of mental health problems. Other work found that reward imbalance is differentially associated with poorer mental health across ethnic groups [53]; however, why Arabic-speaking workers should be most affected in our study is not immediately obvious.

Workers of an Arabic-speaking background were the only group that had significantly poorer mental health compared with Australian-born workers, after adjusting for all job quality factors, employment variables, and sociodemographic characteristics. We collected the data from Arabic-speaking participants in 2015–2016. Since 2001, ethnic groups associated with Islam in Australia experienced increased negative attention from the media, police, security service, and the community in general (Poynting & Mason 2006). While Australia displays Islamophobic tendencies, it also values cultural diversity, which leads to a complex attitude towards migrants from Arabic-speaking countries [54], which may have contributed to the findings we observed in this group.

Our study did not collect information on the type of visa the migrant workers were granted, and it is possible that some results regarding insecurity and unfair pay were the result of “wage theft” or underpayment for work done. A recent report on temporary migrant workers in Australia found that “wage theft” is endemic in this group of workers [45].

The main limitation of this study was that workers of Arabic-speaking, Vietnamese, and Chinese ancestry were sampled differently to the other groups of workers. For the former groups, contact details were obtained from a sample broker, after providing them with a list of the most common surnames among those groups and a list of the ethnically dense suburbs in the cities of Melbourne, Sydney, and Perth. This method resulted in a different demographic of workers being recruited into the survey, when compared with the other two surveys, which used more standard sampling techniques. Quotas were also part of this sampling technique, which meant that not all the provided sample was utilised. Such sampling differences may have led to some of the differences in outcomes we observed between our migrant groups, but it is difficult to know whether this would have over- or underestimated our findings. An unexpected bonus from using this sampling method was that we recruited workers of lower socioeconomic position into the study, e.g., Vietnamese labourers. This highlights the importance of using flexible methods when researching “hard-to-reach” populations.

A second limitation is that we were not able to present the prevalence estimates of job strain (high demand/low control) as we did not collect all the information needed in the first survey to look at job strain in its entirety. Additionally, cut points are often made in terms of percentiles, which work if the sample size is large, but can be very misleading if sample size is small and the numbers do not line up closely with percentiles. For this reason, survey cut points for psychosocial job stressors were estimated separately for each survey.

Strengths of this study include that we have individual-level data collected from workers across the population, rather than focused in high-risk industries. Participants also had the opportunity to complete the telephone survey in their first language, as well as English, thereby not excluding those who may have the greatest risk of exposure.

## 5. Conclusions

This study, the first of its kind, used three cross-sectional surveys to compare and contrast workplace psychosocial stressors and the risk of mental health problems in migrant worker groups in Australia compared with Australian-born workers. With the exception of feeling unfairly paid for work done, all other workplace psychosocial stressors were significantly associated with increased risk of having a mental health problem. Interactions between workplace psychosocial stressors and ethnicity showed variable levels in the risk of having a mental health problem, some attenuated and some increased. The study showed that, while country of birth does play a part in how treatment in the workplace is perceived and responded to, the effects are variable. Putting all groups together as one “migrant worker” group hides these variable effects and highlights the need to include ethnicity as a variable when researching workplace experience in migrant workers. Any interventions to improve workplace conditions for migrant workers also need to be aware of the different experiences related to migrant ethnicity.

## Figures and Tables

**Table 1:** Description of participants’ socio demographic and employment characteristics by country of birth, in the Migrant Workers I and II and Australian Worker surveys.

	Australia *n* = 1062	New Zealand *n* = 566	India *n* = 633	Philippines *n* = 431	Vietnam *n* = 195 *	China *n* = 195 *	Arabic-Speaking *n* = 195 *	All Migrant Workers (*n* = 2215)
Demographics	% (95% CI)	% (95% CI)	% (95% CI)	% (95% CI)	% (95% CI)	% (95% CI)	% (95% CI)	% (95% CI)
Female	46.8 (42.4, 51.2)	36.7 (31.9, 41.7)	37.3 (32.0, 42.9)	43.7 (38.3, 49.2)	46.0 (35.7, 56.7)	51.0 (38.9, 63.0)	35.0 (25.8, 45.5)	40.3 (37.5, 43.1)
Male	53.2 (48.8, 57.6)	63.3 (58.3, 68.1)	62.7 (57.1, 68.0)	56.3 (50.8, 61.7)	54.0 (43.3, 64.3)	49.0 (37.0, 61.1)	65.0 (54.5, 74.2)	59.7 (56.9, 62.5)
Average years lived in Australia	na	19.3 (18.2, 20.5)	12.5 (11.7, 13.3)	15.0 (13.7, 16.3)	22.7 (20.5, 25)	16.5 (14.2, 18.7)	22.4 (20, 24.9)	16.7 (16.1, 17.3)
Aged 18–25 years	15.8 (12.3, 20.1)	11.6 (8.0, 16.6)	10.1 (6.7, 15.0)	9.3 (5.8, 14.6)	5.9 (2.1, 15.8)	9.9 (4.5, 20.1)	18.4 (11.3, 28.3)	10.6 (8.6, 12.9)
Aged 26–35 years	22.9 (18.4, 28.1)	22.2 (17.0, 28.5)	47.3 (41.6, 53.0)	25.7 (20.9, 31.2)	25.1 (15.2, 38.4)	36.1 (24.8, 49.2)	15.6 (9.3, 25.2)	30.4 (27.5, 33.4)
Aged 36–45 years	24.4 (21.1, 28.0)	24.4 (20.1, 29.2)	27.0 (23.2, 31.2)	29.0 (24.5, 34.0)	32.0 (23.2, 42.3)	21.0 (13.0, 32.2)	27.0 (18.0, 38.4)	26.8 (24.5, 29.2)
Aged 46–55 years	23.2 (20.5, 26.2)	24.0 (20.1, 28.4)	10.4 (7.8, 13.7)	22.7 (18.8, 27.1)	28.0 (20.6, 36.9)	24.0 (15.3, 35.6)	25.0 (17.8, 34.0)	20.5 (18.5, 22.6)
Aged 56 years and over	13.7 (11.7, 15.9)	17.8 (14.8, 21.2)	5.2 (4.1, 6.6)	13.3 (10.0, 17.5)	9.0 (6.2, 12.9)	9.0 (5.3, 15.0)	14.0 (8.3, 22.5)	11.8 (10.4, 13.4)
Have up to 12 years school	35.3 (30.9, 40.0)	41.9 (36.2, 47.9)	13.6 (8.6, 21.0)	24.3 (18.8, 30.9)	69.0 (59.3, 77.3)	59.0 (48.2, 69.0)	62.0 (52.7, 70.5)	34.7 (31.6, 37.9)
Have a trade/diploma/certificate	28.1 (24.6, 31.9)	31.0 (26.6, 35.9)	14.0 (10.9, 17.9)	21.0 (17.5, 25.0)	13.0 (7.2, 22.4)	10.0 (6.1, 15.9)	16.0 (10.8, 23.1)	20.1 (18.2, 22.1)
Have tertiary education	36.6 (32.5, 40.9)	27.0 (22.8, 31.7)	72.3 (65.8, 78.0)	54.6 (48.9, 60.2)	18.0 (12.7, 25.0)	31.0 (23.2, 40.1)	22.0 (16.5, 28.8)	45.3 (42.5, 48.1)
Working full-time	58.9 (54.4, 63.3)	74.9 (70.0, 79.3)	71.6 (66.3, 76.5)	66.2 (60.6, 71.4)	73.9 (62.6, 82.7)	65.6 (53.4, 76.1)	64.6 (54.0, 73.9)	70.3 (67.6, 72.9)
	**Australia**	**New Zealand**	**India**	**Philippines**	**Vietnam**	**China**	**Arabic-speaking**	**All migrant workers**
	**% (95% CI)**	**% (95% CI)**	**% (95% CI)**	**% (95% CI)**	**% (95% CI)**	**% (95% CI)**	**% (95% CI)**	**% (95% CI)**
Working part-time	41.1 (36.7, 45.6)	25.1 (20.7, 30.0)	28.4 (23.5, 33.7)	33.8 (28.6, 39.4)	26.1 (17.3, 37.4)	34.4 (23.9, 46.6)	35.4 (26.1, 46.0)	29.7 (27.1, 32.4)
On a casual contract	16.3 (13.0, 20.3)	17.5 (13.6, 22.3)	15.3 (11.7, 19.9)	17.5 (13.4, 22.6)	20.0 (12.1, 31.2)	24.1 (14.8, 36.8)	23.7 (15.7, 34.0)	18.1 (15.8, 20.5)
On a fixed-term contract	8.5 (6.4, 11.2)	5.0 (3.4, 7.5)	7.1 (4.8, 10.4)	4.6 (2.8, 7.5)	2.6 (0.4, 16.3)	13.4 (7.5, 22.6)	3.1 (1.5, 6.2)	5.8 (4.6, 7.2)
On a permanent contract	57.3 (52.8, 61.7)	62.7 (57.2, 67.9)	61.4 (55.6, 67.0)	71.7 (66.3, 76.5)	62.8 (51.8, 72.7)	48.6 (36.6, 60.7)	49.0 (38.7, 59.9)	62.8 (59.9, 65.5)
Self-employed	17.9 (14.8, 21.5)	14.7 (11.3, 18.9)	16.2 (11.8, 21.8)	6.2 (4.3, 8.9)	14.5 (9.4, 21.6)	13.9 (7.6, 24.2)	24.0 (15.9, 34.6)	13.4 (11.5, 15.6)
Average hours per week	35.2 (33.8, 36.6)	39.8 (37.9, 41.8)	35.0 (33.4, 36.5)	35.0 (33.5, 36.4)	34.5 (31.8, 37.1)	32.7 (28.6, 36.9)	34.0 (30.9, 37.2)	36.0 (35.1, 36.9)
Working as managers	8.4 (6.1, 11.6)	10.2 (7.4, 13.9)	10.5 (8.0, 13.7)	8.6 (5.8, 12.5)	4.1 (2.1, 7.9)	7.0 (2.5, 17.8)	8.5 (4.4, 15.7)	9.0 (7.6, 10.7)
Working as professionals	21.2 (17.6, 25.3)	20.2 (16.4, 24.6)	31.3 (26.7, 36.2)	21.4 (17.5, 25.8)	7.5 (4.4, 12.4)	21.7 (14.1, 31.8)	17.4 (11.6, 25.3)	22.4 (20.3, 24.6)
Technicians and trades persons	15.1 (11.8, 19.2)	13.9 (9.9, 19.2)	8.9 (6.5, 12.2)	17.6 (14.1, 21.8)	14.5 (7.9, 25.0)	26.8 (16.5, 40.5)	22.8 (14.7, 33.5)	15.2 (13.1, 17.5)
Community and service workers	12.4 (9.2, 16.5)	8.6 (6.2, 11.8)	8.6 (6.0, 12.2)	9.5 (6.9, 12.9)	19.5 (12.3, 29.5)	16.9 (9.2, 28.9)	15.9 (9.4, 25.5)	10.8 (9.1, 12.6)
Clerical and administrative workers	15.4 (12.4, 18.9)	19.1 (15.1, 23.8)	16.3 (12.5, 21.0)	10.5 (7.7, 14.2)	7.9 (3.4, 17.3)	5.6 (2.6, 11.5)	2.3 (0.6, 7.9)	13.1 (11.3, 15.2)
Sales workers	12.3 (8.8, 16.8)	6.1 (3.9, 9.3)	8.8 (5.8, 13.1)	7.3 (4.5, 11.6)	13.8 (7.4, 24.3)	4.5 (1.6, 12.1)	16.2 (9.6, 26.1)	8.3 (6.7, 10.2)
Machinery operators and drivers	7.0 (4.3, 11.0)	11.2 (8.2, 15.1)	9.1 (5.3, 15.1)	5.7 (3.4, 9.4)	9.5 (5.0, 17.3)	3.5 (1.5, 8.3)	8.2 (3.8, 16.8)	8.3 (6.7, 10.4)
Labourers	8.2 (5.6, 11.9)	10.7 (7.4, 15.2)	6.5 (4.1, 10.3)	19.5 (15.1, 24.8)	23.2 (15.6, 33.1)	14.0 (7.6, 24.4)	8.7 (4.2, 17.3)	12.9 (11.0, 15.1)

* For the age estimate, there were missing values for three migrant groups: Vietnam *n* = 25, China *n* = 26, and Arabic-speaking *n* = 20; na—not applicable.

**Table 2:** Prevalence of psychosocial job quality factors with unadjusted and adjusted odds ratios (aORs) by country of birth, for the Australian Worker, Migrant Worker I, and Migrant Worker II surveys.

	Australia	New Zealand	India	Philippines	Vietnam	China	Arabic-Speaking	All Migrants
Jobs that are complex								
% (95% CI)	24.7 (21.2, 28.7)	26.7 (22.2, 31.8)	25.9 (21.3, 31.1)	27.4 (22.9, 32.4)	30.4 (21.8, 40.5)	17.3 (11.3, 25.5)	25.5 (17.0, 36.3)	26.2 (23.8, 28.7)
OR (95% CI)	1.0	1.1 (0.89, 1.4)	1.0 (0.83, 1.3)	1.1 (0.83, 1.4)	1.6 (1.1, 2.2)	0.81 (0.56, 1.2)	1.0 (0.71, 1.4)	0.38 (0.33, 0.43)
aOR (95% CI)	1.0	1.1 (0.84, 1.4)	0.81 (0.63, 1.1)	1.0 (0.78, 1.4)	2.2 (1.6, 2.9) ***	0.81 (0.59, 1.1)	0.98 (0.72, 1.3)	1.1 (0.87, 1.3)
Jobs with low control								
% (95% CI)	27.6 (23.8, 31.8)	29.2 (24.0, 35.0)	26 (21.5, 31.2)	27.8 (23.0, 33.2)	12.7 (7.0, 22.1)	39.5 (28.2, 52.1)	23.9 (16.2, 33.7)	27.2 (24.6, 29.9)
OR (95% CI)	1.0	1.1 (0.88, 1.4)	1.0 (0.84, 1.3)	0.99 (0.77, 1.3)	0.44 (0.29, 0.68) ***	1.7 (1.2, 2.3) ***	0.87 (0.61, 1.2)	0.9 (0.76, 1.1)
aOR (95% CI)	1.0	0.99 (0.77, 1.3)	1.2 (0.9, 1.5)	0.89 (0.67, 1.2)	0.42 (0.29, 0.6) ***	1.8 (1.3, 2.4) ***	0.94 (0.69, 1.3)	0.98 (0.81, 1.2)
Jobs with low security								
% (95% CI)	34.1 (30.0, 38.5)	32.1 (27.1, 37.6)	32.4 (27.2, 37.9)	31.8 (26.8, 37.3)	34.2 (23.9, 46.2)	41.5 (29.9, 54.1)	30.8 (21.9, 41.4)	32.8 (30.1, 35.7)
OR (95% CI)	1.0	1.1 (0.9, 1.4)	1.5 (1.2, 1.8)	0.9 (0.8, 1.3)	0.9 (0.6, 1.3)	1.7 (1.2, 2.3)	1.1 (0.79, 1.5)	1.2 (1.0, 1.4)
aOR (95% CI)	1.0	1.0 (0.8, 1.3)	1.4 (1.1, 1.8)	0.9 (0.7, 1.2)	0.7 (0.4, 1.0)	1.2 (0.9, 1.8)	0.9 (0.6, 1.3)	1.0 (0.86, 1.3)
Unfair pay								
% (95% CI)	37.0 (32.8, 41.5)	35.2 (30.0, 40.7)	34.8 (29.6, 40.4)	34.0 (28.8, 39.6)	43.4 (31.9, 55.6)	53.7 (41.3, 65.7)	41.0 (31.0, 51.8)	36.0 (33.2, 38.9)
OR (95% CI)	1.0	0.74 (0.6, 0.92)	0.80 (0.65, 0.98)	0.57 (0.44, 0.73)	0.55 (0.39, 0.78)	0.72 (0.52, 1)	0.73 (0.52, 1)	0.59 (0.52, 0.67)
aOR (95% CI)	1.0	0.78 (0.62, 0.99) *	0.95 (0.74, 1.2)	0.60 (0.46, 0.8) ***	0.60 (0.44, 0.81) **	0.79 (0.59, 1.1)	0.77 (0.57, 1)	0.76 (0.64, 0.91)
Overall job adversity								
Two or more measures of adversity								
% (95% CI)	37.0 (32.8, 41.5)	35.2 (30.0, 40.7)	34.8 (29.6, 40.4)	34.0 (28.8, 39.6)	43.4 (31.9, 55.6)	53.7 (41.3, 65.7)	41.0 (31.0, 51.8)	36.0 (33.2, 38.9)
OR (95% CI)	1.0	1.2 (0.94, 1.4)	1.2 (0.95, 1.4)	0.97 (0.76, 1.2)	0.84 (0.58, 1.2)	1.1 (0.79, 1.5)	0.83 (0.6, 1.2)	0.56 (0.49, 0.63)
aOR (95% CI)	1.0	1.1 (0.83, 1.3)	1.2 (0.94, 1.5)	0.89 (0.68, 1.2)	0.74 (0.49, 1.1)	1.0 (0.72, 1.5)	0.81 (0.55, 1.2)	1.0 (0.84, 1.2)

Both the unadjusted and the adjusted odds ratios had the reference group of Australian-born workers. The adjusted odds ratio (aOR) is adjusted for gender, age group, education level, whether or not the job was full-time or part-time, if employment tenure was casual, fixed term, permanent, or the person was self-employed, mean weekly hours worked, and occupation category. Only the adjusted OR was examined for statistically significant differences; * = *p* < 0.05, ** = *p* < 0.001, *** = *p* < 0.0001.

**Table 3:** Risk of having a mental health problem by psychosocial job quality factors and country of birth, Australian Migrant/Ethnic Minority Workers Exposure Survey.

Adjusted ^#^ OR for probability of having a mental health problem by job quality factors				
Job quality factors (reference: not working in those jobs)	**OR**	**95% CI**		***p***
Jobs that are complex or demanding (*n* = 1099)	2.6	2.0	3.4	*p* < 0.0001
Jobs with low control (*n* = 1030)	1.8	1.4	2.4	*p* < 0.0001
Jobs with low security (*n* = 1408)	3.4	2.6	4.4	*p* < 0.0001
Unfair pay (*n* = 1201)	0.9	0.63	1.2	*p* = 0.503
Overall job adversity (*n* = 1135)	2.7	2.0	3.5	*p* < 0.0001
Adjusted ^##^ OR for probability of having a mental health problem by country of birth (reference: Australian born)				
New Zealand (*n* = 633)	1.1	0.6	2.0	*p* = 0.699
India (*n* = 566)	1.4	0.8	2.5	*p* = 0.309
Philippines (*n* = 431)	1.3	0.7	2.5	*p* = 0.405
Vietnam (*n* = 195)	0.9	0.4	2.1	*p* = 0.824
China (*n* = 195)	1.3	0.6	2.6	*p* = 0.541
Arabic-speaking country (*n* = 195)	2.0	1.0	3.9	*p* = 0.052

^#^ Adjusted for gender, age group, education level, mean years lived in Australia, employment status and employment type, mean hours worked weekly, and occupational category. ^##^ Adjusted for workplace psychosocial stressors, gender, age group, education level, mean years lived in Australia, employment status, employment type, and occupation.

**Table 4:** The likelihood of having a mental health problem by the interaction between country of birth and psychosocial job quality in the Migrant Worker I and Migrant Worker II surveys with Australian-born workers as the reference group.

Measures of Adverse Psychosocial Job Quality	New Zealand	India	Philippines	Vietnam	China	Arabic-Speaking	All Migrants
Jobs that are complex							
OR ^a^ (95% CI)	0.95 (0.46, 2)	1.2 (0.61, 2.3)	1.7 (0.77, 3.7)	1.9 (0.58, 6.3)	0.72 (0.25, 2.0)	1.4 (0.55, 3.5)	1.2 (0.71, 1.9)
aOR ^b^ (95% CI)	1.0 (0.47, 2.3)	1.2 (0.57, 2.4)	1.6 (0.68, 3.7)	2.2 (0.5, 10.0)	0.68 (0.21, 2.3)	1.6 (0.52, 5.1)	1.2 (0.68, 2.1)
Jobs with low control							
OR ^a^ (95% CI)	1.4 (0.68, 3.0)	1.2 (0.61, 2.3)	0.72 (0.31, 1.7)	6.4 (1.9, 2.1)	0.63 (0.24, 1.7)	1.1 (0.4, 2.8)	1.2 (0.7, 2.0)
aOR ^b^ (95% CI)	1.4 (0.63, 3.2)	1.4 (0.64, 2.9)	0.65 (0.25, 1.7)	8.1 (1.8, 37)	0.73 (0.23, 2.3)	1.5 (0.45, 4.7)	1.2 (0.68, 2.2)
Jobs with low security							
OR ^a^ (95% CI)	1.0 (0.49, 2.2)	1.1 (0.55, 2.2)	1.1 (0.49, 2.5)	0.50 (0.13, 2.0)	0.27 (0.1, 0.70)	0.56 (0.23, 1.4)	0.83 (0.5, 1.4)
aOR ^b^ (95% CI)	1.1 (0.48, 2.5)	1.1 (0.52, 2.4)	1.3 (0.55, 3.2)	0.25 (0.04, 1.5)	0.27 (0.08, 0.85) *	0.59 (0.19, 1.8)	0.89 (0.48, 1.6)
Unfair pay							
OR ^a^ (95% CI)	1.3 (0.61, 2.6)	1.6 (0.82, 3)	0.9 (0.41, 2)	2.1 (0.65, 6.5)	0.61 (0.23, 1.7)	1.4 (0.56, 3.5)	1.2 (0.76, 2.0)
aOR ^b^ (95% CI)	0.73 (0.23, 2.3)	0.48 (0.13, 1.7)	2.6 (0.9, 7.7)	3.3 (0.72, 15.0)	0.96 (0.23, 3.9)	4.3 (1.2, 15.0) *	1.4 (0.59, 3.1)
Overall job adversity							
OR ^a^ (95% CI)	1.0 (0.46, 2.2)	0.86 (0.44, 1.7)	0.83 (0.37, 1.9)	2.7 (0.51, 1.4)	0.38 (0.15, 1.0)	0.79 (0.31, 2.0)	0.83 (0.49, 1.4)
aOR ^b^ (95% CI)	1.1 (0.49, 2.6)	0.94 (0.44, 2.0)	0.84 (0.35, 2.0)	2.1 (0.36, 1.2)	0.35 (0.11, 1.1)	0.87 (0.28, 2.7)	0.89 (0.48, 1.6)

^a^ OR is the unadjusted odds ratio of having a mental health problem by the interaction between job quality factors and country of birth, with the reference group as Australian-born workers. ^b^ Adjusted OR (aOR) is the odds ratio of having a mental health problem by the interaction of job quality factors and country of birth adjusted for gender, age group, education level, mean years in Australia, employment status, employment type, mean weekly hours worked, and occupation, with the reference group as Australian-born workers; * *p* < 0.05.

## Supplementary Materials

The following are available online at https://www.mdpi.com/1660-4601/16/5/735/s1, Table S1: Distribution of the migrant survey respondents by source of sample and country of birth.
