# Supplementary material for: Using Three Cross-Sectional Surveys to Compare Workplace Psychosocial Stressors and Associated Mental Health Status in Six Migrant Groups Working in Australia Compared with Australian-Born Workers
Source: Int J Environ Res Public Health. 2019 Feb 28;16(5):735. doi: 10.3390/ijerph16050735 (PMC6427607; doi:10.3390/ijerph16050735)
Supplement: Supplementary file 1 [file ijerph-16-00735-s001.pdf]

Supplementary Table 1. Distribution of the migrant survey respondents by source of sample and country of birth.

Sample source	New Zealand	India	Philippines
EWP stratified random - surname based	280	319	146
EWP random migrant dense suburbs - surname based which were mostly in metropolitan areas	140	97	54
Sample broker (unknown sources)	122	196	217
Other (advertising and snowballing)	24	21	14
Total sample recruited	566	633	431
